# Biologics protect psoriasis patients from being exacerbated by COVID-19 infection

**DOI:** 10.1016/j.heliyon.2024.e24534

**Published:** 2024-01-14

**Authors:** Yu-Xin Zheng, Xi-Bei Chen, Zhao-Yuan Wang, Li-Ran Ye, Min Zheng, Xiao-Yong Man

**Affiliations:** Department of Dermatology, Second Affiliated Hospital, Zhejiang University School of Medicine, Hangzhou, China

**Keywords:** Psoriasis, Biologics, COVID-19, OAS

## Abstract

**Background:**

Patients with psoriasis may experience an exacerbation in symptoms following COVID-19 infection. After abandoning 'zero COVID' strategies, China experienced a surge of Omicron infections.

**Objectives:**

We aimed to investigate psoriasis exacerbation in psoriatic patients with COVID-19, following treatment with three different biologics, adalimumab, secukinumab, and ixekizumab.

**Methods:**

We performed a prospective study (n = 209) at our hospital between November 01, 2022, and February 15, 2023. We defined △ PASI as post-COVID-19 PASI minus pre-COVID-19 PASI. Two endpoints were set in this study. △ PASI >0 was defined as exacerbation of psoriasis after infection. △ PASI >3 was defined as a severe exacerbation of psoriasis symptoms after infection. In addition, serum OAS1, OAS2, and OAS3 were also assessed.

**Results:**

Results showed that the severity of psoriasis can worsen after COVID-19 infection, and a smaller proportion of patients taking biologics developed worsening psoriasis compared to those not using biologics; however, only the patients taking ixekizumab demonstrated a statistically significant difference (p < 0.05), while those taking adalimumab or secukinumab didn't. What's more, the use of biological agents suppressed the serum OAS2 and OAS3 at low levels and elevated the serum OAS1 level in patients with psoriasis.

**Conclusions:**

This study provided new insights into the protective role of biological agents in patients with psoriasis who were infected with COVID-19, and we proposed that psoriatic patients treated with biologics should continue with the treatment during the COVID-19 pandemic.

## Abbreviations

SARS-CoV-2: severe acute respiratory syndrome coronavirus 2
COVID-19: Corona Virus Disease 2019
CPRWERG: Chinese Psoriasis Real World Evidence Research Group
PASI: psoriasis area and severity index
ADA: Adalimumab
IXE: Ixekizumab
SEC: Secukinumab
OAS: 2′-5′-oligoadenylate synthetase
OAS1: 2′-5′-Oligoadenylate Synthetase 1
OAS2: 2′-5′-Oligoadenylate Synthetase 2
OAS3: 2′-5′-Oligoadenylate Synthetase 3
PBMCs: peripheral blood mononuclear cells
ELISA: enzyme-linked immunosorbent assay

## 1 Introduction

Psoriasis is an immune-mediated chronic inflammatory disease [1]. During the COVID-19 pandemic, cases of vaccination causing inflammatory skin manifestations, including psoriasis, were reported globally [2,3]. Clinicians also reported cases of worsening psoriasis caused by COVID-19 infection [4,5]. At the same time, scholars have suggested that certain biologics may be beneficial for psoriasis patients with COVID-19 infection [6,7]. Currently, biologics targeting TNF, IL-17, and IL-12/23, including etanercept, adalimumab (ADA), secukinumab (SEC), ixekizumab (IXE), brodalumab, stekinumab, and guselkumab, are widely used in clinical practice for managing psoriasis [8]. During the COVID-19 pandemic, a relatively large number of psoriasis patients at our center were undergoing treatment with ADA, SEC, or IXE. To the best of our knowledge, none of the studies have reported on the control of psoriasis severity in patients with COVID-19 infection receiving these biologics.

After abandoning ‘zero COVID’ strategies on December 7, 2022, China experienced a surge in Omicron infections [9]. The purpose of this study was to characterize whether there are differences in the severity of psoriasis in patients using biologics following COVID-19 infection.

Numerous studies have demonstrated the close relationship between the 2′-5′-oligoadenylate synthetase (OAS) family and COVID-19 infections [[10], [11], [12], [13]]. Members of the OAS family, OAS1, OAS2, and OAS3, play a vital role in limiting viral infections by activating Rnase L to restrict viral replication [14]. Thus, serum markers of psoriasis patients using different biologics, including OAS1, OAS2, and OAS3, were also tested.

## 2 Materials and methods

### 2.1 Study design and population

This study was conducted at the Second Affiliated Hospital of Zhejiang University, a tertiary hospital in China. The study enrolled 209 patients (>18 years of age) who presented to dermatology clinics with plaque psoriasis between November 01, 2022, and February 15, 2023. Only psoriatic patients who had severe acute respiratory syndrome coronavirus 2 (SARS-CoV-2) infection confirmed using a positive polymerase chain reaction test or positive Antigen test were included in the analysis. All patients were treated with or without biologic agents for psoriasis before they were infected with COVID-19. The non-biologics included topical therapies and phototherapy. Three biological agents were evaluated in this study: ADA, IXE, and SEC. In addition, enrolled patients using biologics had achieved PASI75 or above before COVID-19 infection and did not receive biologics during COVID-19 infection, with the aim of minimizing changes in Psoriasis Area and Severity Index (PASI) scores due to therapeutic resistance or biologics injection.

This study was conducted in accordance with the principles of the Declaration of Helsinki and approved by the Second Affiliated Hospital of Zhejiang University Human Research Ethics Board (2020-485). Written informed consent was obtained from all participating individuals.

### 2.2 Baseline assessment and follow-up

Clinical variables were collected and included demographics, current treatments, comorbidities, family history of psoriasis, baseline PASI, and body surface area (BSA) before using biologics, as well as PASI score before COVID-19 infection. PASI score was recorded again within two weeks of a patient's confirmed COVID-19 infection.

We defined △ PASI as post-COVID-19 PASI minus pre-COVID-19 PASI. Two endpoints were set in this study. △ PASI >0 was defined as exacerbation of psoriasis after infection. △ PASI >3 was defined as a severe exacerbation of psoriasis symptoms after infection. Clinical data were extracted from the CPRWERG (Chinese Psoriasis Real World Evidence Research Group), and the accuracy was validated using electronic medical records.

### 2.3 Serum collection and ELISA for OASs

Further, we collected serum from patients with psoriasis (n = 176) who had COVID-19 infections within two weeks after undergoing different treatments. The patients were divided into four groups: the non-biological agents, ADA, SEC, and IXE. The expression levels of serum OAS1, OAS2, and OAS3 were detected using an enzyme-linked immunosorbent assay (ELISA) (LSBio, Seattle, WA, catalog number LS-F31934, LS-F8120, LS-F34102). ELISA was carried out in accordance with the manufacturer's instructions.

### 2.4 Statistical methods and data analysis

Statistical analysis was performed using SPSS 27.0 software. Normality was tested using the Shapiro–Wilk test. Normally distributed or approximately normally distributed data are presented as mean ± SD ($\bar{x}\pm s$). For multigroup comparisons, one-way ANOVA tests were performed, and the least significant difference test was performed for comparison between the two groups. Non-normal distribution or rank data are represented by the median and quartile M (P25, P75). Pair-wise comparisons were made using Wilcoxon rank-sum, while multiple group data comparisons were made using Kruskal–Wallis tests with Bonferroni-corrected post-hoc test between groups. Counting data are represented by the number of cases and rates (n, %), and the comparison of rates was based on the chi-square test. The statistical graph was created using GraphPad Prism 9. For all analyses, α = 0.05 was set for statistical significance.

## 3 Results

### 3.1 Characteristics of psoriatic patients under different treatments

Demographic and clinical characteristics of enrolled patients are shown in Table 1. Patients were divided into four groups: non-biologics (n = 28), ADA (n = 23), IXE (n = 94), and SEC (n = 64). The mean age was 40.74 ± 14.06 years, and the mean duration of psoriasis was 10 years (range 5–17 years). Specific details for the four groups are shown in Supplementary Table 1. Significantly more patients had arthritis in the ADA group than in the other three groups (p < 0.001). The average psoriasis course was also an incomparable factor (P = 0.039). The mean psoriasis duration in the non-biologics group was shorter than that in the SEC group (8.5(2.25,10) and 10(7.25,20), p < 0.05). Other data, including age, sex, BMI, family history of psoriasis, and complications of cardiovascular disease, were comparable between the groups.

**Table 1:** Demographic and clinical characteristics of psoriatic patients.

Characteristic	$\bar{x}\pm$ s/n (%)/*M (P*_25_, *P*_75_)
Age, y	40.74 ± 14.06
Male sex	138 (66)
BMI, kg/m^2^	24.23 ± 4.14
Course of psoriasis, y	10 [5,15]
Current treatment	
non-biologics	28 (13.4)
Adalimumab	23(11)
Ixekizumab	94 (45)
Secukinumab	64 (30.6)
Family history of psoriasis	9 (4.3)
Arthritis	26 (12.4)
Cardiovascular disease	9 (4.3)
Baseline PASI	12 (6.5,18)
Baseline BSA	15 [8,16]

Note: BMI, Body mass index; y, year; PASI, Psoriasis area and severity index; BSA, Body surface area.

### 3.2 Median PASI of psoriatic patients increased following COVID-19 infection

We compared PASI before and after COVID-19 infection in patients of different treatment groups (Table 2). In the non-biologics group, PASI increased from 4.5 (3, 11.6) to 6.7 (3.23, 12.55) (p = 0.002); in the ADA group, PASI increased from 1.4 (0, 3.2) to 2.2 (0.5, 3.6) (p = 0.041), in the IXE group, PASI increased from 0.3 (0, 0.98) to 0.4 (0, 1.2) (p = 0.035), and in the SEC group, PASI increased from 0.6 (0, 1.93) to 1.2 (0.6, 3) (p = 0.007). Therefore, all groups showed a statistically significant increase in median PASI after COVID-19 infection.

**Table 2:** PASI before and after COVID-19 infection in the four different treatment groups.

Index	None (n = 28)	ADA (n = 23)	IXE (n = 94)	SEC (n = 64)
PASI before COVID-19 infection	4.5(3,11.6)	1.4(0,3.2)	0.3(0,0.98)	0.6(0,1.93)
PASI after COVID-19 infection	6.7(3.23,12.55)	2.2(0.5,3.6)	0.4(0,1.2)	1.2(0.6,3)
Z-score	3.028	2.040	2.109	2.674
*P*-value	0.002	0.041	0.035	0.007

Note: Wilcoxon rank-sum was used to compare PASI before and after COVID-19 infection in each group.

Abbreviations: PASI, Psoriasis area and severity index; ADA, adalimumab; IXE, ixekizumab; SEC, secukinumab.

### 3.3 Patients treated with IXE were less likely to experience psoriasis exacerbation following COVID-19 infection

Taking △PASI >0 as the indicator of psoriasis aggravation, the proportion of patients who experienced psoriasis aggravation in the non-biologics, ADA, IXE, and SEC groups was 57.1 %, 34.8 %, 18.1 %, and 37.5 %, respectively (p = 0.001). Similarly, when △PASI >3 was considered as an indicator of severe psoriasis exacerbation, the proportion of patients who experienced psoriasis exacerbation in the non-biologics, ADA, IXE, and SEC groups was 21.4 %, 13.0 %, 3.2 %, and 9.4 %, respectively (p = 0.019) (Table 3). At both endpoints, the proportion of patients who experienced psoriasis exacerbation in the three biologics-treated groups was lower than that in the non-biologics group, but only the IXE group showed a statistically significant difference (p < 0.05). This suggested that IXE may be a better biologic for protecting psoriasis patients from disease exacerbation after COVID-19 infection.

**Table 3:** Aggravation of psoriasis following COVID-19 infection in treatment groups with different biological agents.

	None (n = 28)	ADA (n = 23)	IXE (n = 94)	SEC (n = 64)	χ^2^	*P value*
△PASI >0	16(57.1)^a^	8(34.8)^a,b^	17(18.1)^b^	24(37.5)^a^	17.662	0.001
△PASI >3	6(21.4)^a^	3(13)^a,b^	3(3.2)^b^	6(9.4)^a,b^	9.974	0.019

Note: Different letters indicate statistically significant differences (p < 0.05). The Chi-square test was used to compare the proportion of △PASI >0 or △PASI >3 between the four different groups. Abbreviations: PASI, Psoriasis area and severity index; ADA, adalimumab; IXE, ixekizumab; SEC, secukinumab.

As for △PASI >0, IXE and SEC, both targeting IL-17A, showed a significant difference in patient outcome (p < 0.05), suggesting that IXE was better than SEC in controlling psoriasis following COVID-19 infection. However, there was no statistical difference between the two groups for △PASI >3.

### 3.4 Biological agents suppress the serum OAS2 and OAS3 at low levels and elevate the serum OAS1 level in patients with psoriasis

**Fig. 1:**
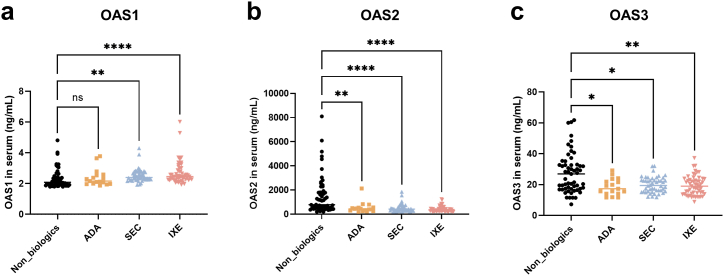
Serum OAS1, OAS2, and OAS3 levels of psoriasis patients infected with COVID-19 in different treatment groups. (a–c) OAS1, OAS2, and OAS3 levels in serum (ng/mL) were detected in the non-biological agent (n = 55), ADA (n = 17), SEC (n = 48), and IXE (n = 56) groups. Statistical analysis of the data was performed using the Kruskal–Wallis tests. (ns, P > 0.05; *P < 0.05; **P < 0.01; ****P < 0.0001). ADA, adalimumab; IXE, ixekizumab; SEC, secukinumab.

Subsequently, we collected serum from patients with psoriasis (n = 176) who had COVID-19 infections within two weeks after undergoing different treatments. The patients were divided into four groups: the non-biological agents (n = 55), ADA (n = 17), SEC (n = 48), and IXE (n = 56). We found that serum OAS1 was higher in the SEC and IXE groups than in the non-biological agent group (P < 0.01 and P < 0.0001, respectively) (Fig. 1a). Meanwhile, serum OAS2 was significantly lower in all the patients treated with biological agents than in those treated with non-biological agents (Fig. 1b) (P < 0.01, P < 0.0001, P < 0.0001, respectively). Similarly, OAS3 levels were lower in the biological agent groups (Fig. 1c) (P < 0.05, P < 0.05, P < 0.01, respectively).

## 4 Discussion

The COVID-19 pandemic has had a global impact on the management of psoriasis and various other diseases [4,17,18]. Discontinuation of medications due to COVID-19 has the potential to adversely affect the outcomes of skin diseases [15]. At the beginning of the COVID-19 epidemic, there was concern that psoriatic patients receiving biologics would be more susceptible to COVID-19 due to the immunomodulatory effects [19]. The National Psoriasis Foundation suggested that psoriatic patients not infected with SARS-CoV-2 should continue their biological therapies [17]. Similarly, a single-center study from Turkey, in which all psoriatic patients were on a given biologic agent, suggested that biologics for psoriasis did not cause an additional risk in patients infected with COVID-19 [20].

Mounting evidence suggests that the use of IL-17 inhibitors, including SEC, IXE, and brodalumab in patients with psoriasis does not increase the risk of SARS-CoV-2 infection or worsen the course of COVID-19 [21,22]. In addition, IXE, SEC, and brodalumab can reduce the mRNA level of SARS-CoV-2 receptor ACE2 in skin lesions of patients with psoriasis [23,24]. IXE can also reduce the ACE2 protein level in psoriatic skin lesions [25]. SEC and IXE, the two biologics targeting IL-17A, are often compared, whereas their IgG subclasses differ [26,27]; the former is an IgG1 monoclonal antibody, and the latter is an IgG4 monoclonal antibody [26,27]. In addition, real-world studies of psoriasis patients have shown that IXE users have better treatment adherence and persistence, as well as a lower risk of discontinuation compared with SEC users [28,29]. Another one-year real-world study conducted in Spain showed no differences between SEC and IXE in biologics-naïve or biologics-experienced patients, but a higher PASI 75 response was observed at week 52 for IXE users previously treated with two or more biologics [30].

Clinicians also reported a case of a patient with psoriasis and psoriatic arthritis who initiated ADA treatment before contracting COVID-19 and experienced a rapid recovery from the infection [31]. This may be attributed to the immunomodulatory effects that can reduce the intensity of the immune response, thereby mitigating the severity of cytokine release syndrome and improving the overall prognosis [16].

In our study, IXE outperformed SEC and ADA in protecting against psoriasis exacerbation during COVID-19 infection. Specifically, the proportion of psoriatic patients experiencing psoriasis aggravation following COVID-19 infection was lower in the IXE group than that in the other groups.

We conducted this study to aid dermatologists in determining the optimal way to treat psoriatic patients during the COVID-19 pandemic. We observed that psoriasis severity can worsen with COVID-19 infection. Notably, our results showed that treatment with IXE decreased the proportion of psoriatic patients who experienced worsening disease compared with patients who did not use any biological agent. In our study, biologics-naïve patients with aggravated psoriasis following COVID-19 infection were advised to use biologics to control the disease. If the patient was using ADA, SEC, or IXE, the original biologic dose was increased, or the biologic treatment was switched. For patients who did not experience psoriasis worsening, the original treatment was continued.

OAS1, OAS2, and OAS3 are part of the interferon-induced genes which have been equivalent to an anti-viral function [14]. Since the OAS proteins are expressed constitutively at low levels, they are regarded as pattern-recognition receptors for the detection of viral RNA [32]. Our previous study [33] showed that OAS2 and OAS3 were elevated in the serum of psoriatic patients and lower after SEC treatment. This study suggested that during the COVID-19 pandemic, ADA, SEC, and IXE may suppress serum OAS2 and OAS3 levels in patients with psoriasis, thereby preventing psoriasis exacerbation. Single-cell RNA sequencing of peripheral blood mononuclear cells (PBMCs) from COVID-19 cohorts also showed that OASs were downregulated in monocytes after corticosteroid treatment [34].

In summary, this study provides new insights into the protective role of biological agents in patients with psoriasis who were infected with COVID-19. We proposed that psoriatic patients treated with biological agent therapy should continue with the treatment during the COVID-19 pandemic. Furthermore, IXE may be a better therapeutic option than ADA and SEC for treating psoriatic patients in the COVID-19 pandemic. In addition, the use of biological agents may suppress the serum OAS2 and OAS3 at low levels and elevate the serum OAS1 level in patients with psoriasis. Confirmatory studies are needed to evaluate the generalizability of our findings.

### 4.1 Limitations of the study

This study had some limitations. First, the sample size was small. Second, it was challenging to rule out other reasons for elevated PASI scores besides COVID-19 infection, such as instances when biologics could not be administered as planned during the COVID-19 pandemic. Moreover, our study exclusively included outpatients, potentially limiting its representativeness of patients who required hospitalization.

## 5 Conclusions

In summary, this study provides new insights into the protective role of biologics in individuals with psoriasis who contracted COVID-19. Specifically, COVID-19 infection exacerbated the severity of psoriasis. A lower percentage of psoriasis patients using biologics experienced deterioration compared to those not using biologics. IXE exhibited a statistically significant difference, whereas individuals using ADA or SEC did not, but the proportion of aggravated patients showed a decreasing trend. Additionally, the utilization of biological agents resulted in the suppression of serum OAS2 and OAS3 at low levels, along with an elevation in the serum OAS1 level among patients with psoriasis.

## Bulleted statements

### What is already known about this topic?

In the COVID-19 pandemic, biologics might be protective and beneficial for patients with psoriasis. OASs are part of the interferon-induced genes which have been equivalent to an anti-viral function.

### What does this study add?

Ixekizumab may be a better therapeutic option than adalimumab and secukinumab for treating psoriatic patients in the COVID-19 pandemic. In addition, the use of biological agents suppressed the serum OAS2 and OAS3 and elevated the serum OAS1 level in psoriasis patients.

## Ethics statement

This study was in accordance with the principles of the Declaration of Helsinki and approved by the Second Affiliated Hospital of Zhejiang University Human Research Ethics Board (2020-485). Written informed consent was obtained from all participants.

## Data availability statement

No datasets were generated or analyzed during the current study. Data included in article/supp. Data is available on request from the authors.

## Funding sources

This work was supported by grants from the 10.13039/501100014857National Natural Science Foundation of China (Nos. 82230104, 82073426, 82373466).

## CRediT authorship contribution statement

**Yu-Xin Zheng:** Writing - review & editing, Writing - original draft, Project administration, Investigation, Formal analysis. **Xi-Bei Chen:** Writing - review & editing, Writing - original draft, Validation, Project administration, Investigation. **Zhao-Yuan Wang:** Writing - original draft, Project administration, Investigation. **Li-Ran Ye:** Writing - review & editing, Investigation, Data curation. **Min Zheng:** Writing - review & editing, Funding acquisition, Conceptualization. **Xiao-Yong Man:** Writing - review & editing, Supervision, Project administration, Methodology, Funding acquisition, Conceptualization.

## Declaration of competing interest

The authors declare that they have no known competing financial interests or personal relationships that could have appeared to influence the work reported in this paper.
