# Supplementary material for: Biologics protect psoriasis patients from being exacerbated by COVID-19 infection
Source: Heliyon. 2024 Jan 14;10(2):e24534. doi: 10.1016/j.heliyon.2024.e24534 (PMC10828055; doi:10.1016/j.heliyon.2024.e24534)
Supplement: Supplementary file 1 [file mmc1.docx]

Supplementary Material

# Supplementary Table

Supplementary Table 1. Demographic and clinical characteristics of patients with different treatments.

| Characteristic | None (n=28) | ADA (n=23) | IXE (n=94) | SEC (n=64) | F/H/χ^2^ | *P value* |
| --- | --- | --- | --- | --- | --- | --- |
| Age, y | 34(27,54) | 41(35,60) | 38(29,47.25) | 39.5(27,52.75) | 5.077 | 0.166 |
| BMI, kg/m^2^ |  |  |  |  | 11.851 | 0.198 |
| BMI＜18.5 | 2(7.1) | 4(17.4) | 9(9.6) | 3(4.7) |  |  |
| 18.5≤BMI＜24.9 | 17(60.7) | 15(65.2) | 47(50) | 29(45.3) |  |  |
| 25≤BMI＜29.9 | 8(28.6) | 4(17.4) | 26(27.7) | 23(35.9) |  |  |
| BMI≥30 | 1(3.6) | 0(0) | 12(12.8) | 9(14.1) |  |  |
| Male sex | 16(57.1) | 13(56.5) | 65(69.1) | 44(68.8) | 2.532 | 0.470 |
| Course of psoriasis, y | 8.5(2.25,10)^b^ | 10(4,25)^a,b^ | 10(4,17)^a,b^ | 10(7.25,20)^a^ | 8.373 | 0.039 |
| Family history of psoriasis | 0(0) | 2(8.7) | 4(4.3) | 3(4.7) | 2.233 | 0.479 |
| Arthritis | 2^a^(7.1) | 13^b^(56.5) | 5^a^(5.3) | 6^a^(9.4) | 32.208 | <0.001 |
| Cardiovascular disease | 0(0) | 2(8.7) | 2(2.1) | 5(07.8) | 4.909 | 0.128 |

Note: Different letters indicate statistically significant differences ( p＜0.05). One-way ANOVA was applied to evaluate differences between BMI among different groups. Age and course of psoriasis were analyzed by the Kruskal-Wallis H rank sum test. Chi-square test was used for other indexes. BMI, Body mass index; y, year.
